# Editorial: Methods and Applications in Implementation Science

**DOI:** 10.3389/fpubh.2019.00213

**Published:** 2019-07-31

**Authors:** Mary E. Northridge, Donna Shelley, Thomas G. Rundall, Ross C. Brownson

**Affiliations:** ^1^Hansjörg Wyss Department of Plastic Surgery, NYU School of Medicine, New York University (NYU) Langone Dental Medicine - Brooklyn, Brooklyn, NY, United States; ^2^Department of Population Health, NYU School of Medicine, New York, NY, United States; ^3^The Center for Lean Engagement and Research (CLEAR), University of California, Berkeley School of Public Health, Berkeley, CA, United States; ^4^Prevention Research Center in St. Louis, Brown School at Washington University in St. Louis, St. Louis, MO, United States; ^5^Division of Public Health Sciences, Department of Surgery, Alvin J. Siteman Cancer Center, Washington University School of Medicine, St. Louis, MO, United States

**Keywords:** adaptation, evaluation, frameworks, implementation strategies, measurement, mechanisms, partnerships, sustainability

In a classic review, Green et al. popularized the pipeline graphic that depicts the 17-year odyssey necessary for the production and transfer of knowledge from research to practice and policy (1). Still, the vetting of research through successive scientific filters does little to assure that the populations in need of evidence-based practices ever benefit from scientific advances. This Research Topic is intended to provide insights from implementation science that move beyond the clinical care of individual patients, to also take account of provider, organizational, systems, and policy levels pertaining to health and health care.

Testable theories that describe the causal pathways through which implementation strategies effect change are needed to improve the outcomes produced by evidence-based interventions (EBIs). Lewis et al. advance an innovative four-step approach to building causal pathway models that articulates the mediators, moderators, preconditions, and proximal and distal outcomes of implementation processes. Such clarity in causal pathways will allow us to understand better where, when, and why strategies have an effect on outcomes of interest.

The RE-AIM framework (2) provides important guidance for planning and assessing dimensions that influence the implementation process and potential for EBIs to impact population health. Harden et al. articulate how an updated RE-AIM framework addresses emerging implementation science priorities, such as cost and adaptation, and includes a greater focus on contextual and explanatory factors. Powell et al. present a research agenda for five priorities that need to be addressed to increase the public health impact of implementation strategies: (1) enhance methods for designing and tailoring; (2) specify and test mechanisms of change; (3) conduct more effectiveness research on discrete, multifaceted, and tailored strategies; (4) increase economic evaluations; and (5) improve tracking and reporting. For economic evaluations, the range of approaches is vast, from simple costing to full cost-effectiveness analyses. Okamura et al. report on an innovative method for calculating training and consultation costs related to delivering evidence-based treatments (EBT) that may provide insight into how systems should prioritize training efforts.

Partnerships, engagement, and collaboration (PEC) are important strategies for advancing dissemination and implementation of EBIs in clinical and community settings, but conceptual models and methods to guide design and evaluation of PECs is lacking. Huang et al. conducted a scoping review of the PEC literature that identified key domains, processes, mechanisms, and strategies for PEC, and proposed a new multilevel framework to guide future research in this area. Mazzucca et al. assessed the research designs and methodologies used in 212 dissemination and implementation (D&I) study protocols recently published in *Implementation Science*. While a large majority of the protocols (77%) utilized randomized designs, and most protocols (61%) proposed quantitative and qualitative methods, only 52% reported using a theoretical framework to guide the study. Northridge et al. present a protocol for a participatory, multilevel, dynamic intervention to improve the oral health of low-income Chinese Americans, guided by two complementary, multilevel frameworks: Consolidated Framework for Implementation Research (CFIR) (3) and Implementation Outcomes Framework (IOF) (4). Lee et al. utilized a novel multiphase, explanatory sequential mixed methods design to provide deeper understanding of how complex multisector partnerships impact population health outcomes in an evaluation of the Massachusetts Prevention and Wellness Trust Fund.

As per the public health adage, “what gets measured gets done,” (5) progress in implementation requires the development of practical measures that are both reliable and valid. Budd et al. developed and tested a tool for measuring the contextual factors related to evidence-based practice across four countries (Australia, Brazil, China, and the United States), and found variability in reliability across domain and country, suggesting that some items are highly generalizable, while others are less so. Dearing conducted a review of 30 available organizational readiness tools, noting that even as most measure capacity, few measure organizational motivation. Helfrich et al. assessed organizational readiness to change over two waves in a workplace health promotion trial, and found that change commitment declined significantly at both intervention and control sites over time, even as wellness-program effort increased significantly at intervention sites.

Adapting EBIs to the local context is a necessary step to facilitate adoption and implementation. Approaches are needed that promote a systematic approach to documenting and evaluating the adaptation process. Rabin et al. make an important contribution by describing a multilevel, multimethod adaptation approach across four health systems, guided by the Stirman framework (6) for adaptation and modification and expanded using concepts from the RE-AIM framework (2). The modified adaptation model showed promise in capturing adaptation across a range of projects and content areas. To scale-up an evidence-based parenting program for prevention of pediatric obesity, Smith et al. report on the multiyear process of adaptation to a new clinical target and service delivery system. In a study of behavioral health treatment, Patel et al. apply an instructional design framework in the development and evaluation of e-learning modules as either a single component or one strategy in a multifaceted approach for training in evidence-based practices (EBPs).

Detailed specification of implementation strategies is a challenge, especially for complex, multilevel interventions that use multiple strategies. Huynh et al. describe a five-step method for mapping intervention strategies and demonstrate its use with a study of the implementation of a cardiovascular toolkit. Fernandez et al. introduce Implementation Mapping, which provides a systematic process for developing strategies to improve the adoption, implementation, and maintenance of evidence-based interventions in real-world settings. Pullmann et al. report on findings from a study of the impact of clinical supervision to improve the adoption of EBT for child mental health problems. Findings point to the importance of a supportive organizational climate in predicting supervisory EBT intensity.

Brookman-Frazee et al. contribute to the limited research on EBP sustainment in mental health services long after implementation, illustrating a novel application of survival analysis to administrative claims data in system-driven implementation of multiple EBPs. Finally, Palinkas et al. point to opportunities for using agency leader models to develop strategies to facilitate implementation of evidence-based and innovative practices for children and adolescents, guided by the Stages of Implementation Completion framework (7). Our hope is that this collection advances the field.

## Author Contributions

All authors listed have made a substantial, direct and intellectual contribution to the work, and approved it for publication.

### Conflict of Interest Statement

The authors declare that the research was conducted in the absence of any commercial or financial relationships that could be construed as a potential conflict of interest.
